# Comparison of Maternal–Fetal Outcomes among Unvaccinated and Vaccinated Pregnant Women with COVID-19

**DOI:** 10.3390/jpm12122008

**Published:** 2022-12-03

**Authors:** Alicia Martínez-Varea, Elena Satorres, Sandra Florez, Josep Domenech, Julia Desco-Blay, Sagrario Monfort-Pitarch, María Hueso, Alfredo Perales-Marín, Vicente Diago-Almela

**Affiliations:** 1 Department of Obstetrics and Gynaecology, La Fe University and Polytechnic Hospital, Avenida Fernando Abril Martorell 106, 46026 Valencia, Spain; 2 Department of Economics and Social Sciences, Universitat Politècnica de València, Camí de Vera s/n, 46022 Valencia, Spain

**Keywords:** COVID-19, SARS-CoV-2 variants, pregnancy, vaccination

## Abstract

**Background**: This study sought to elucidate whether COVID-19 vaccination, during gestation or before conception, entails a decreased incidence of severe COVID-19 disease during pregnancy. **Methods**: This retrospective cohort study included all pregnant women that were followed up at a tertiary University Hospital with SARS-CoV-2 infection diagnosed between 1 March 2020 and 30 July 2022. The primary outcome of the study was to compare maternal and perinatal outcomes in unvaccinated and vaccinated pregnant patients with SARS-CoV-2 infection. **Results**: A total of 487 pregnant women with SARS-CoV-2 infection were included. SARS-CoV-2 infection during the third trimester of pregnancy was associated with an 89% lower probability of positive cord-blood SARS-CoV-2 IgG antibodies (OR 0.112; 95% CI 0.039–0.316), compared with infection during the first or the second trimester. Vaccinated pregnant women (201 (41.27%)) with COVID-19 had an 80% lower risk for developing pneumonia and requiring hospital admission due to COVID-19 than unvaccinated patients (aOR 0.209; 95% CI 0.044–0.985). Noticeably, pregnant patients with SARS-CoV-2 infection with at least two doses of the COVID-19 vaccine did not develop severe COVID-19. **Conclusion**: Vaccinated women with SARS-CoV-2 infection during pregnancy are associated with decreased hospital admission due to COVID-19 as well as reduced progression to severe COVID-19.

## 1. Introduction

Severe acute respiratory syndrome coronavirus 2 (SARS-CoV-2) was first identified in Wuhan, China, in December 2019 [1]. Due to its notably contagious behavior [2], over 629 million people have been infected, and over 6,5 million have died worldwide as of 27 October 2022 [3]. Mainly transmitted through respiratory droplets [4], the novel coronavirus leads to fever, cough [4,5], and pneumonia [4,6].

Coronavirus Disease 2019 [7] during pregnancy is associated with an increased risk of severe disease [8,9,10] as well as pregnancy complications [8,9,11]. Indeed, compared with nonpregnant women with COVID-19, pregnant patients are more prone to require admission into the intensive care unit (ICU) [10,12,13,14], oxygen therapy [13], invasive ventilation [10,12,13,14], and extracorporeal membrane oxygenation [12] and are more susceptible to death [12,14]. Moreover, particularly severe COVID-19 during pregnancy is associated with low birthweight [15], preterm birth [8,14,15,16,17,18], preeclampsia [8,15,16,19], and stillbirth [8,15,16,17]. Thus, COVID-19 vaccination has been strongly recommended for pregnant women to protect mothers [9,10,20,21] and infants [9,22]. Preliminary data are reassuring regarding the safety of the COVID-19 vaccine for pregnant and lactating women [10,17,21,23,24,25,26]. Recent reports reveal that COVID-19 vaccines are not associated with detrimental consequences on pregnancy outcomes [7,20,21,23,27,28,29]. Nonetheless, data are still limited. Additionally, studies are scant concerning the effectiveness and immune response of COVID-19 vaccines on pregnant women [10,17] and the transfer of IgG antibodies to fetuses [10,26]. Hence, this study sought to elucidate whether COVID-19 vaccination, during gestation or before conception, entails a decreased incidence of severe COVID-19 disease during pregnancy and pregnancy complications associated with COVID-19.

## 2. Materials and Methods

### 2.1. Study Design and Participants

This retrospective cohort study included all pregnant women that were followed up at the tertiary University and Polytechnic Hospital La Fe (Valencia, Spain) with SARS-CoV-2 infection diagnosed between 1 March 2020 and 30 July 2022. Patients were included if the diagnosis of SARS-CoV-2 infection was performed during pregnancy. Women were excluded from the study if they had an ongoing pregnancy as of 30 July 2022, were lost to follow-up, or were aged below 18 years of age.

The diagnosis of SARS-CoV-2 infection was based on a positive result on real-time reverse-transcription polymerase chain reaction (RT-PCR) assay [5] or rapid antigen test [30] of nasopharyngeal swab specimens. Pregnant women were tested at the hospital or at the outpatient clinics due to symptoms of, or exposure to, the virus at the time of triage. All pregnant women admitted to the hospital during the study period were also tested. Moreover, pregnant women who reported a positive rapid antigen test performed at home or at the pharmacy because of symptoms of, or exposure to, the SARS-CoV-2 were also included in the study. Neonates of pregnant women who were positive for SARS-CoV-2 during pregnancy or at the time of the delivery were tested with RT-PCR or cord-blood SARS-CoV-2 immunoglobulin G (IgG) and IgM antibodies within 24 hours after delivery.

Data regarding COVID-19 infection during pregnancy, history of COVID-19 vaccination, and maternal and perinatal outcomes were collected from the digital clinical history of the hospital. All medical records were anonymized. Ethical approval for the study was obtained from the Ethical Committee of the Health Research Institute Hospital La Fe (IIS La Fe).

### 2.2. Outcomes

The outcomes of the study were, firstly, to compare maternal and perinatal outcomes of pregnant patients with asymptomatic and symptomatic SARS-CoV-2 infection. Secondly, to compare maternal and perinatal outcomes in unvaccinated and vaccinated pregnant patients with SARS-CoV-2 infection. Finally, to analyze the severity of COVID-19 disease among pregnant women according to the different SARS-CoV-2 variants and vaccination status.

Symptomatic SARS-CoV-2 infection was defined by the presence of symptoms that included fever, cough, dyspnea, and ageusia-anosmia [4,5,6], associated with a positive RT-PCR assay or rapid antigen test. Fever was defined as an axillary temperature of 38 °C or higher. Asymptomatic SARS-CoV-2 infection was determined by the absence of symptoms associated with a positive RT-PCR assay or rapid antigen test. Vaccinated individuals were defined as those who have received at least one dose of COVID-19 vaccine before the SARS-CoV-2 infection. Obesity was defined as a body mass index ≥ 30 kg/m^2^. Gestational age was established according to the first-trimester ultrasound. Miscarriage was defined as pregnancy loss prior to 22 weeks of gestation and stillbirth as fetal demise at or beyond 22 weeks. Neonatal death was defined as the death of a liveborn infant in the first 28 days postpartum. Small for gestational age (SGA) was defined as an ultrasound-estimated fetal weight between the 10th and the 3rd percentile with normal Doppler [31,32]. Fetal growth restriction (FGR) was determined by an estimated fetal weight below the 3rd percentile or by the combination of an estimated fetal weight below the 10th percentile with a cerebroplacental ratio beneath the 5th percentile or a mean pulsatility index of the uterine arteries above the 95th percentile [32,33]. Chronic hypertension was defined by systolic blood pressure ≥140 mmHg and diastolic blood pressure ≥90 mmHg before pregnancy or before 20 weeks of gestation [34]. Preeclampsia was defined by new-onset hypertension (repeated measurement of systolic blood pressure ≥140 mmHg and diastolic blood pressure ≥90 mmHg) after 20 weeks of pregnancy and the coexistence of one or both of the following new-onset conditions: proteinuria (urine protein:creatinine ratio ≥30 mg/mmol, or albumin:creatinine ratio ≥8 mg/mmol, or ≥1 g/L [2+] on dipstick testing, or 300 mg protein in a 24-hour urine collection), or other maternal organ dysfunction, including renal, liver, neurological, or hematological complications, or uteroplacental dysfunction (such as fetal growth restriction, abnormal umbilical artery Doppler waveform analysis, or stillbirth) [35,36,37,38]. Gestational diabetes mellitus was defined by fasting plasma glucose >126 mg/dL, casual plasma glucose >200 mg/dl, or plasma or serum glucose >140 mg/dl 1 hour after a 50-gram glucose challenge test followed by at least two abnormal glucose values in the three hours and 100-gram oral glucose tolerance test (≥105 mg/dL, 1 hour ≥190 mg/dL, 2 hours ≥165 mg/dL, and 3 hours ≥145 mg/dL) [39,40]. Preterm premature rupture of membranes was defined as spontaneous rupture of the membranes before 37 weeks of gestation [41]. Threatened preterm labor was defined as the presence of uterine contractions and shortening of the uterine cervix [42]. Preterm birth was defined as delivery before 37^+0^ weeks of gestation [43].

Four different timeframes of variants’ predominance were identified based on the Spanish Ministry of Health data [44]: timeframe 1, when the Pre-alpha variants prevailed, included cases until 2 April 2021; timeframe 2, when the Alpha variant prevailed in Spain, until 6 August 2021; timeframe 3, when the Delta variant was predominant, until 21 December 2021; and, afterward, timeframe 4 included the cases when Omicron was the principal variant in Spain.

### 2.3. Statistical Analysis

Categorical variables were presented as absolute and relative frequencies. Continuous variables were shown as means and standard deviations. Comparisons between the characteristics of the groups were performed using Student’s *t*-test or Kruskal–Wallis test for continuous variables and Fisher’s exact testing for categorical variables.

To assess the potential association between the symptoms and the perinatal outcomes, a multivariate logistic regression was performed adjusting for nulliparity, pre-existing chronic diseases, advanced maternal age (≥36 years old), obesity, and smoking habit. A secondary analysis was also performed to determine the vaccination effects on outcomes, considering the same confounders. Another multivariate logistic regression was performed to assess whether the diagnosis of COVID-19 in the third trimester was associated with the presence of cord-blood SARS-CoV-2 IgG antibodies, adjusting for symptoms and vaccination status. Adjusted odds ratios with a 95% confidence interval were calculated. Analyses were performed in R version 4.0.3.

## 3. Results

A total of 487 pregnant women that were followed up at our institution had COVID-19 during the study period. Characteristics of pregnant patients with symptomatic (n = 287) and asymptomatic (n = 200) SARS-CoV-2 infection were compared. A higher prevalence of pregnant women through *in vitro* fertilization among symptomatic patients (9.8% *versus* 3%, *p* = 0.004) was found. No other statistical differences were found between groups. Additionally, whereas the majority of pregnant women with COVID-19 during the second trimester (149/487 [30.6%]) were symptomatic (38% *versus* 20%, *p* < 0.001), a larger proportion of patients who tested positive for SARS-CoV-2 infection during the third trimester of pregnancy (279/487 [57.3%]) were asymptomatic (50.2% *versus* 67.5%, *p* < 0.001) (Table 1).

Regarding maternal outcomes of symptomatic and asymptomatic pregnant women with COVID-19, no patients with asymptomatic SARS-CoV-2 infection developed pneumonia, required hospital admission due to COVID-19, needed antibiotics, corticosteroids, antiviral drugs, or oxygen therapy, or had a composite adverse maternal outcome (Table 2). Among the 28/287 pregnant women with symptomatic COVID-19 who developed pneumonia, one was diagnosed with an associated pulmonary embolism. There were no maternal deaths in our cohort. In respect of perinatal outcomes, newborns from pregnant women with symptomatic COVID-19 displayed a decreased proportion of negative cord-blood SARS-CoV-2 IgG antibodies, compared to newborns from asymptomatic patients (26.8% *versus* 48.8%, *p* = 0.045). IgM was not detected in any cord-blood sample. Interestingly, only four newborns from pregnant patients with a symptomatic SARS-CoV-2 infection had a positive SARS-CoV-2 RT-PCR within 24 hours after delivery (Table 2). These three pregnant women, two with a singleton pregnancy and one with a twin pregnancy, had COVID-19 at the time of the delivery. Parameters for perinatal outcomes assessed with a multivariable logistic regression were not statistically significant (Table 3).

**Figure 1:**
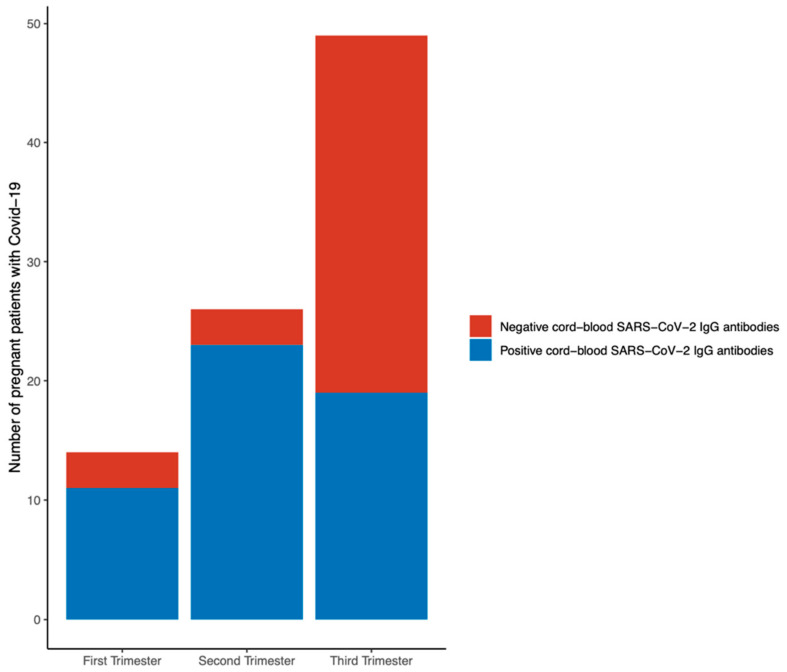
Cord-blood SARS-CoV-2 IgG antibody seropositivity of newborns from pregnant women with SARS-CoV-2 infection during the indicated trimester of pregnancy.

A logistic regression demonstrated that SARS-CoV-2 infection during the third trimester of pregnancy was associated with an 89% lower probability of a positive cord-blood SARS-CoV-2 IgG antibodies (odds ratio (OR) 0.112; 95% confidence interval (CI) 0.039–0.316), compared with SARS-CoV-2 infection during the first or the second trimester of pregnancy. When controlled for vaccination status and symptoms during SARS-CoV-2 infection, the adjusted odds ratio (aOR) is 0.115, 95% CI 0.040–0.3331 (Figure 1).

**Figure 2:**
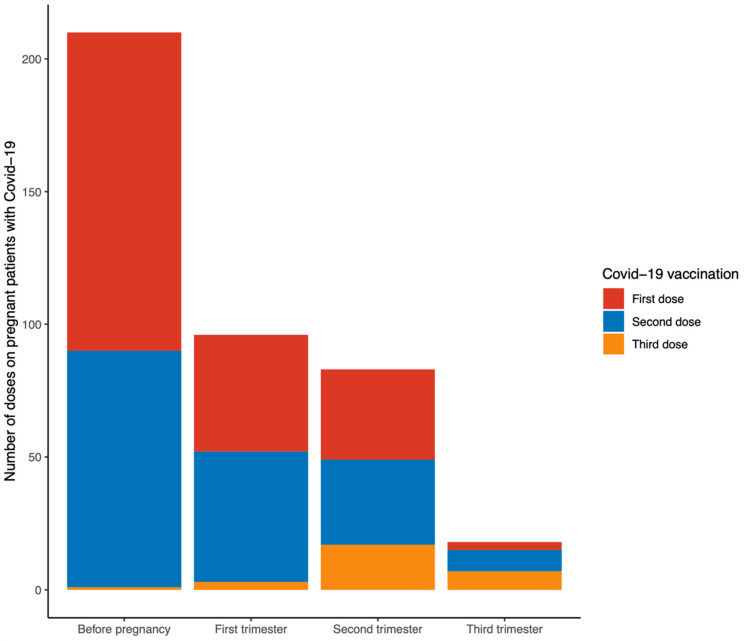
Timing of COVID-19 vaccination in the pregnant patients (201/487 (41.27%)) who received at least one COVID-19 vaccine before the SARS-CoV-19 infection. The number of patients that received the first, second, or third dose of a vaccine before or during the different trimesters of pregnancy is shown.

Whereas 201/487 (41.27%) pregnant patients received at least one COVID-19 vaccine before the SARS-CoV-19 infection, 286/487 (58.73%) patients were not vaccinated before the diagnosis of COVID-19. A total of 128/201 (63.7%) patients received a COVID-19 vaccine during pregnancy. While 81/128 received the first COVID-19 dose during gestation, 47/128 were vaccinated during pregnancy and before conception. The patients that were only vaccinated before pregnancy were 73/128 (Figure 2). Vaccinated patients prior to COVID-19 had a higher age (33.0 *versus* 31.2 years, *p* < 0.001), a larger requirement of assisted reproductive techniques (11.9% *versus* 4.2%, *p* = 0.002), particularly *in vitro* fertilization (10.9% *versus* 4.2%, *p* = 0.006), a reduced incidence of SARS-CoV-2 infection during the first trimester (5.5% *versus* 16.8%, *p* < 0.001), and a higher incidence of SARS-CoV-19 infection during the second trimester of pregnancy (36.8% *versus* 26.2%, *p* = 0.016), compared with the non-vaccinated pregnant women with COVID-19 (Table 4).

**Figure 3:**
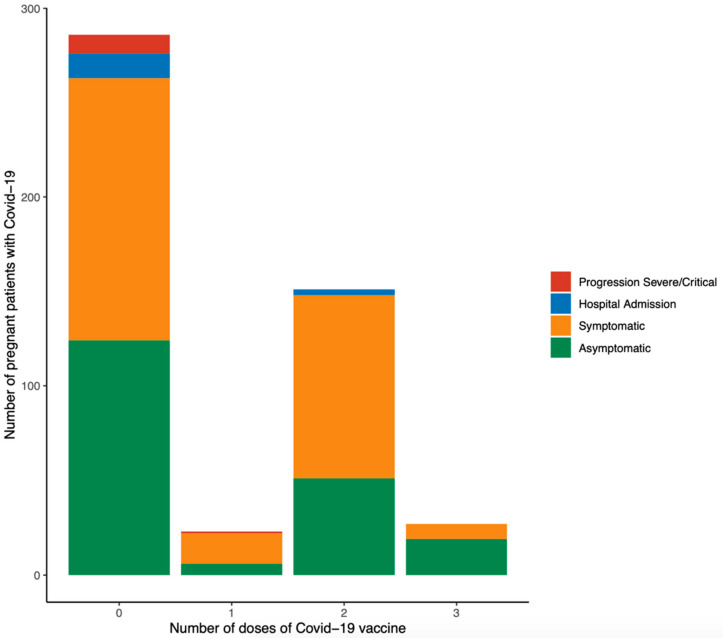
Progression to severe COVID-19 among vaccinated pregnant women with SARS-CoV-2 infection according to the number of doses of COVID-19 vaccine. Severe COVID-19 was defined as at least one of the following: admission to the intensive care unit, intubation, or extracorporeal membrane oxygenation.

**Figure 4:**
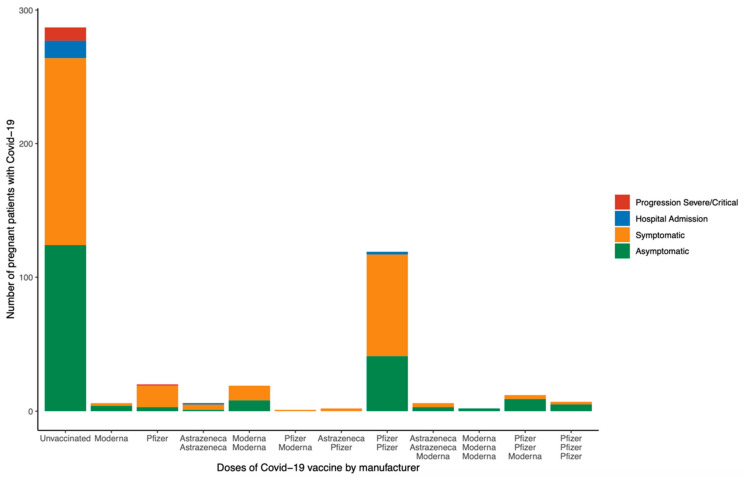
Progression to severe COVID-19 among vaccinated pregnant women with SARS-CoV-2 infection according to the number of doses and type of COVID-19 vaccine. Severe COVID-19 was defined as at least one of the following: admission to the intensive care unit, intubation, or extracorporeal membrane oxygenation.

In respect of maternal outcomes of vaccinated and non-vaccinated pregnant patients with COVID-19, vaccinated women associated a significantly reduced incidence of pneumonia (2.0% *versus* 8.4%, *p* = 0.03), hospital admission due to COVID-19 (2.0% *versus* 8.0%, *p* = 0.004), composite adverse maternal outcome (0.5% *versus* 3.5%, *p* = 0.031), and necessity of antibiotics (0.5% *versus* 4.2%, *p* = 0.019), corticosteroids (0.5% *versus* 4.5%, *p* = 0.01), and oxygen therapy (0.5% *versus* 5.2%, *p* = 0.003; Table 5). Pregnant patients with SARS-CoV-2 infection with at least two doses of the COVID-19 vaccine did not develop severe COVID-19 (Figure 3 and Figure 4). Perinatal outcomes were not significantly different between vaccinated and unvaccinated pregnant patients with COVID-19 (Table 5). A multivariate logistic regression demonstrated that vaccinated pregnant women with COVID-19 had an 80% lower risk for developing pneumonia and requiring hospital admission due to COVID-19 than unvaccinated patients (aOR 0.209; 95% CI 0.044–0.985). Other parameters concerning perinatal outcomes addressed within the logistic regression model were not statistically significant (Table 6).

**Figure 5:**
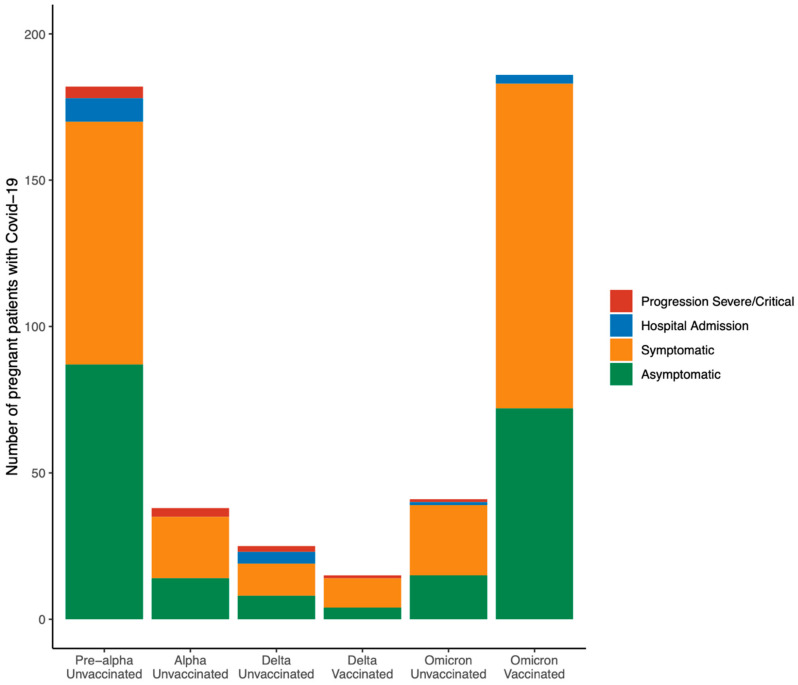
Progression to severe COVID-19 among vaccinated pregnant women with SARS-CoV-2 infection according to the SARS-CoV-2 strain predominance and vaccination status. Severe COVID-19 was defined as at least one of the following: admission to the intensive care unit, intubation, or extracorporeal membrane oxygenation.

Different variants of SARS-CoV-2 were predominant during the study period. In this cohort, all pregnant women with COVID-19 when the Pre-alpha and Alpha strains were predominant in Spain were unvaccinated. Noticeably, unvaccinated pregnant patients with COVID-19 when the Delta strain prevailed in Spain had the highest rate of hospital admission (24.0%) and the highest progression to severe COVID-19 disease (8.0%) compared to patients in the Pre-alpha period (6.6% and 2.2%, respectively), patients when the Alpha strain was predominant (all 7.9% patients who required hospital admission developed severe COVID-19), and unvaccinated patients when the Omicron strain was dominant (4.8% and 2.4%, respectively). Regarding vaccinated patients when Omicron prevailed in Spain, only 1.6% required hospital admission, and none underwent severe COVID-19 disease (Figure 5).

## 4. Discussion

The main findings of this retrospective cohort study that included 487 pregnant women with COVID-19 include: (1) asymptomatic patients did not develop pneumonia and did not require hospital admission due to COVID-19; (2) SARS-CoV-2 infection during the third trimester of pregnancy was associated with an 89% lower probability of positive cord-blood SARS-CoV-2 IgG antibodies (OR 0.112; 95% CI 0.039–0.316), compared with the infection during the first or the second trimester of pregnancy; (3) vaccinated pregnant women (201 (41.27%)) with COVID-19 had an 80% lower risk for developing pneumonia and requiring hospital admission due to COVID-19 than unvaccinated patients (aOR 0.209; 95% CI 0.044–0.985); (4) pregnant patients with SARS-CoV-2 infection with at least two doses of the COVID-19 vaccine did not develop severe COVID-19; and (5) unvaccinated pregnant patients with COVID-19 when the Delta strain prevailed in Spain had the highest rate of hospital admission (24.0%), compared to unvaccinated patients when other strains were predominant.

It has been described that physiological, immunologic, and mechanical changes during gestation could predispose to COVID-19 [8,45,46]. Nonetheless, data are scarce to conclude whether or not pregnancy raises vulnerability to SARS-CoV-2 [8]. In the present study, asymptomatic pregnant patients with SARS-CoV-2 infection did not develop pneumonia and did not require hospital admission due to COVID-19. These findings are in concordance with those described by the World Association of Perinatal Medicine Working Group on COVID-19, which reported that SARS-CoV-2 infection during gestation was associated with an 11.1% rate of admission into the intensive care unit, which was significantly higher in symptomatic pregnant women [47]. It has also been reported that, compared with asymptomatic pregnant patients with SARS-CoV-2 infection, those with severe disease had an increased risk of perinatal complications, such as hypertensive disorders of pregnancy, preterm birth, and cesarean delivery [48]. The present study did not find significant differences regarding perinatal complications between asymptomatic and symptomatic pregnant patients with SARS-CoV-2 infection. It might be due to the limited sample size or a higher proportion of vaccinated pregnant women. Interestingly, newborns from pregnant women with symptomatic COVID-19 analyzed in the present study displayed a decreased proportion of negative cord-blood SARS-CoV-2 IgG antibodies, compared with newborns from asymptomatic patients (26.8% *versus* 48.8%, *p* = 0.045). Nonetheless, a study with a higher sample size has shown that IgG antibodies to SARS-CoV-2 are present in the cord blood of newborns from symptomatic as well as asymptomatic pregnant women with SARS-CoV-2 [49]. These cord-blood antibody concentrations have been correlated with maternal antibody titles [49] and with the duration between infection and delivery [49,50]. In concordance, in the present study, SARS-CoV-2 infection during the third trimester of pregnancy was associated with an 89% lower probability of positive cord-blood SARS-CoV-2 IgG antibodies irrespective of vaccination status and symptoms during SARS-CoV-2 infection. Given that other authors have reported that the titles of anti-Spike antibodies found in cord blood are lower after SARS-CoV-2 infection during gestation than with COVID-19 vaccination in pregnancy [51,52], further studies are required in order to clarify neonatal protection according to the trimester of both SARS-CoV-2 infection and COVID-19 vaccination. In contrast, only four newborns from this cohort of pregnant patients with a symptomatic SARS-CoV-2 infection at the time of delivery had a positive SARS-CoV-2 RT-PCR within 24 hours after delivery. This finding is in concordance with the reportedly infrequent intrauterine SARS-CoV-2 transmission [8].

In this cohort, 41.27% of pregnant women with COVID-19 had received at least one COVID-19 vaccine before SARS-CoV-2 infection. These patients had an 80% lower risk of developing pneumonia and requiring hospital admission due to COVID-19 than unvaccinated patients (aOR 0.209; 95% CI 0.044–0.985). Noticeably, pregnant patients with SARS-CoV-2 infection with at least two doses of the COVID-19 vaccine did not develop severe COVID-19. It has been reported that a significant proportion of pregnant women are reluctant to receive the first COVID-19 vaccination [23,53] or a booster [54] during gestation. Nonetheless, not only does current data show the safety of COVID-19 vaccination during pregnancy [10,17,21,23,24,25,26], but it also shows that it does not entail adverse pregnancy outcomes [7,20,21,23,27,29]. Thus, COVID-19 vaccination has been strongly recommended for pregnant women to protect both mothers [9,10,20,21,55] and infants [9,22]. COVID-19 vaccine types are those using mRNA (Pfizer, Moderna), viral vector (such as J&J Janssen or AstraZeneca), and inactivated [56]. COVID-19 vaccines using mRNA have been the most widely used in pregnant women and are associated with the most extensive accumulated safety data as well as effectiveness against COVID-19 [56]. Therefore, COVID-19 mRNA vaccines have been recommended as the first option for pregnant women [56]. Despite the limited sample size of the present study, pregnant patients with SARS-CoV-2 infection that have previously received two doses of AstraZeneca displayed the highest hospital admission rate due to COVID-19 compared to pregnant patients with SARS-CoV-2 infection that have previously received two COVID-19 vaccines using mRNA or one AstraZeneca and the other using mRNA.

Unvaccinated pregnant patients with COVID-19 when the Delta strain prevailed in Spain had the highest rate of hospital admission (24.0%) and the highest progression to severe COVID-19 disease (8.0%), compared to unvaccinated patients when other strains were predominant. These results are consistent with the previously reported increased severity of COVID-19 in pregnant women during the Delta variant predominance [57,58,59,60]. Moreover, in the present study, the vaccinated pregnant women with SARS-CoV-2 infection when the Delta variant prevailed were associated with decreased hospital admission and disease severity, similar to previous reports [57,61]. Regarding vaccinated patients when Omicron prevailed in Spain, only 1.6% required hospital admission, and none underwent severe COVID-19 disease. These findings concord with the reported decreased disease severity associated with Omicron [57,62,63].

Future studies are required to determine the optimal timing of COVID-19 vaccination during pregnancy for maternal and neonatal benefit [21]. Additionally, further studies are needed in order to confirm whether SARS-CoV-2 infection during the first and the second trimester is associated with a higher vertical transfer of SARS-CoV-2 IgG antibodies, compared with the infection during the third trimester of pregnancy.

The main strength of the present study is the analysis of maternal and perinatal outcomes of all pregnant women with SARS-CoV-2 infection from a tertiary hospital during the study period, regardless of the trimester of diagnosis and pregnancy outcome. The study also compared maternal and perinatal outcomes among vaccinated and unvaccinated pregnant patients with SARS-CoV-2 infection during the different variants of COVID-19 and associated the number and type of COVID-19 vaccines received prior to the infection. Drawbacks of the study include the limited sample size, as well as the restricted number of newborns from pregnant women with COVID-19 that were tested with RT-PCR or cord-blood SARS-CoV-2 IgG and IgM antibodies within 24 hours after delivery. Additionally, the variants of SARS-CoV-2 were established according to the Spanish Ministry of Health data (44) and not by sequencing analysis.

## 5. Conclusions

Vaccinated pregnant women with SARS-CoV-2 infection display an 80% lower risk of developing pneumonia and requiring hospital admission due to COVID-19 than unvaccinated patients. Pregnant patients with SARS-CoV-2 infection who had previously received at least two doses of the COVID-19 vaccine did not develop severe COVID-19. Hence, this study further encourages COVID-19 vaccination prior to conception or during pregnancy.

## Figures and Tables

**Table 1:** Pregnant women with SARS-CoV-2 infection, overall and according to the presence of symptoms.

	Total Sample (n = 487)	Symptomatic (n = 287)	Asymptomatic (n = 200)	*p*
Maternal age (years)	32.0 ± 5.5	32.0 ± 5.7	31.9 ± 5.3	0.96
Pre-existing chronic disease *	93/486 (19.1 (15.7–22.9))	58/286 (20.3 (15.8–25.4))	35/200 (17.5 (12.5–23.5))	0.483
Smoking habit	32/485 (6.6 (4.6–9.2))	21/286 (7.3 (4.6–11.0))	11/199 (5.5 (2.8–9.7))	0.463
Weight (kg)	66.4 ± 14.1	65.7 ± 14.2	67.4 ± 14.0	0.213
Obesity **	47/338 (13.9 (10.4–18.1))	23/200 (11.5 (7.4–16.8))	24/138 (17.4 (11.5–24.8))	0.15
Nulliparity	240/483 (49.7 (45.1–54.2))	147/283 (51.9 (46.0–57.9))	93/200 (46.5 (39.4–53.7))	0.268
Twin pregnancy	16/487 (3.3 (1.9–5.3))	9/287 (3.1 (1.4–5.9))	7/200 (3.5 (1.4–7.1))	0.803
Assisted reproductive techniques	36/487 (7.4 (5.2–10.1))	28/287 (9.8 (6.6–13.8))	8/200 (4.0 (1.7–7.7))	0.021
Artificial insemination	2/487 (0.4 (0.0–1.5))	0/287 (0.0 (0.0–1.3))	2/200 (1.0 (0.1–3.6))	0.168
*In vitro* fertilization	34/487 (7.0 (4.9–9.6))	28/287 (9.8 (6.6–13.8))	6/200 (3.0 (1.1–6.4))	0.004
Egg donation	4/487 (0.8 (0.2–2.1))	4/287 (1.4 (0.4–3.5))	0/200 (0.0 (0.0–1.8))	0.148
SARS-CoV-2 infection during 1st trimester	59/487 (12.1 (9.4–15.3))	34/287 (11.8 (8.3–16.2))	25/200 (12.5 (8.3–17.9))	0.888
SARS-CoV-2 infection during 2nd trimester	149/487 (30.6 (26.5–34.9))	109/287 (38.0 (32.3–43.9))	40/200 (20.0 (14.7–26.2))	<0.001
SARS-CoV-2 infection during 3rd trimester	279/487 (57.3 (52.8–61.7))	144/287 (50.2 (44.2–56.1))	135/200 (67.5 (60.5–73.9))	<0.001

Data are given as n/N (%) or mean ± SD. * Including diabetes, chronic hypertension, asthma, autoimmune disease, infection by human immunodeficiency virus, hepatitis B virus, or hepatitis C virus. ** Defined as body mass index ≥ 30 kg/m^2^.

**Table 2:** Maternal and perinatal outcomes of 487 pregnancies with SARS-CoV-2 infection, overall and according to the presence of symptoms.

	Total Sample (n = 487)	Symptomatic (n = 287)	Asymptomatic (n = 200)	*p*
**Maternal outcome**				
Pneumonia	28/487 (5.7 (3.9–8.2))	28/287 (9.8 (6.6–13.8))	0/200 (0.0 (0.0–1.8))	<0.001
Hospital admission due to COVID-19	27/487 (5.5 (3.7–8.0))	27/287 (9.4 (6.3–13.4))	0/200 (0.0 (0.0–1.8))	<0.001
Admission into the intensive care unit	11/27 (40.7 (22.4–61.2))	11/27 (40.7 (22.4–61.2))	0/0 (0.0 (0.0–0.0))	1.0
Composite adverse maternal outcome *	11/487 (2.3 (1.1–4.0))	11/287 (3.8 (1.9–6.8))	0/200 (0.0 (0.0–1.8))	0.004
Antibiotic treatment	13/487 (2.7 (1.4–4.5))	13/287 (4.5 (2.4–7.6))	0/200 (0.0 (0.0–1.8))	0.001
Corticosteroid drug	14/487 (2.9 (1.6–4.8))	14/287 (4.9 (2.7–8.0))	0/200 (0.0 (0.0–1.8))	<0.001
Antiviral drug	10/487 (2.1 (1.0–3.7))	10/287 (3.5 (1.7–6.3))	0/200 (0.0 (0.0–1.8))	0.007
Oxygen therapy	16/487 (3.3 (1.9–5.3))	16/287 (5.6 (3.2–8.9))	0/200 (0.0 (0.0–1.8))	<0.001
Intubation	7/487 (1.4 (0.6–2.9))	7/287 (2.4 (1.0–5.0))	0/200 (0.0 (0.0–1.8))	0.045
Extracorporeal membrane oxygenation	1/487 (0.2 (0.0–1.1))	1/287 (0.3 (0.0–1.9))	0/200 (0.0 (0.0–1.8))	1.0
**Perinatal outcome**				
Ectopic pregnancy	1/487 (0.2 (0.0–1.1))	0/287 (0.0 (0.0–1.3))	1/200 (0.5 (0.0–2.8))	0.411
Miscarriage	9/487 (1.8 (0.8–3.5))	4/287 (1.4 (0.4–3.5))	5/200 (2.5 (0.8–5.7))	0.498
Stillbirth	2/487 (0.4 (0.0–1.5))	1/287 (0.3 (0.0–1.9))	1/200 (0.5 (0.0–2.8))	1.0
***Completed pregnancies (excluding ectopic pregnancy and miscarriage)***				
Threatened preterm labor	5/477 (1.0 (0.3–2.4))	3/283 (1.1 (0.2–3.1))	2/194 (1.0 (0.1–3.7))	1.0
Small for gestational age	4/477 (0.8 (0.2–2.1))	1/283 (0.4 (0.0–2.0))	3/194 (1.5 (0.3–4.5))	0.309
Fetal growth restriction	16/477 (3.4 (1.9–5.4))	10/283 (3.5 (1.7–6.4))	6/194 (3.1 (1.1–6.6))	1.0
Gestational diabetes mellitus	30/477 (6.3 (4.3–8.9))	21/283 (7.4 (4.7–11.1))	9/194 (4.6 (2.1–8.6))	0.253
Gestational hypertension	6/477 (1.3 (0.5–2.7))	1/283 (0.4 (0.0–2.0))	5/194 (2.6 (0.8–5.9))	0.043
Preeclampsia	4/477 (0.8 (0.2–2.1))	2/283 (0.7 (0.1–2.5))	2/194 (1.0 (0.1–3.7))	1.0
Preterm premature rupture of membranes	3/477 (0.6 (0.1–1.8))	1/283 (0.4 (0.0–2.0))	2/194 (1.0 (0.1–3.7))	0.569
Gestational age at delivery (weeks)	39.2 ± 1.7	39.2 ± 1.8	39.1 ± 1.6	0.168
Cesarean section	121/477 (25.4 (21.5–29.5))	74/283 (26.1 (21.1–31.7))	47/194 (24.2 (18.4–30.9))	0.669
Preterm birth	38/475 (8.0 (5.7–10.8))	20/282 (7.1 (4.4–10.7))	18/193 (9.3 (5.6–14.3))	0.393
*Singleton pregnancy*				
Gestational age at delivery (weeks)	39.3 ± 1.7	39.3 ± 1.8	39.2 ± 1.5	0.187
Preterm birth	29/459 (6.3 (4.3–8.9))	16/273 (5.9 (3.4–9.3))	13/186 (7.0 (3.8–11.7))	0.697
Birth weight (g)	3254.2 ± 480.6	3263.3 ± 500.4	3240.9 ± 451.0	0.483
1 min Apgar	9.2 ± 1.1	9.2 ± 1.0	9.2 ± 1.2	0.717
5 min Apgar	9.9 ± 0.5	9.9 ± 0.5	9.9 ± 0.4	0.602
5 min Apgar < 7	2/458 (0.4 (0.1–1.6))	2/273 (0.7 (0.1–2.6))	0/185 (0.0 (0.0–2.0))	0.517
Umbilical artery pH	7.2 ± 0.1	7.2 ± 0.1	7.3 ± 0.1	0.202
Umbilical artery pH < 7.1	6/447 (1.3 (0.5–2.9))	3/269 (1.1 (0.2–3.2))	3/178 (1.7 (0.3–4.8))	0.686
Umbilical venous pH	7.3 ± 0.1	7.3 ± 0.1	7.3 ± 0.1	0.254
Neonatal morbidity **	43/459 (9.4 (6.9–12.4))	28/273 (10.3 (6.9–14.5))	15/186 (8.1 (4.6–13.0))	0.515
Neonatal death	1/459 (0.2 (0.0–1.2))	1/273 (0.4 (0.0–2.0))	0/186 (0.0 (0.0–2.0))	1.0
Positive RT-PCR SARS-CoV-2	2/122 (1.6 (0.2–5.8))	0/48 (0.0 (0.0–7.4))	2/74 (2.7 (0.3–9.4))	0.519
Positive cord-blood SARS-CoV-2 immunoglobulin G antibodies	52/84 (61.9 (50.7–72.3))	30/41 (73.2 (57.1–85.8))	22/43 (51.2 (35.5–66.7))	0.045
*Twin pregnancy*				
Gestational age at delivery (weeks)	36.4 ± 0.9	36.7 ± 0.9	36.0 ± 0.9	0.135
Preterm birth	9/16 (56.3 (29.9–80.2))	4/9 (44.4 (13.7–78.8))	5/7 (71.4 (29.0–96.3))	0.358
Birth weight (g) of the first twin	2408.6 ± 363.0	2441.7 ± 364.2	2366.0 ± 385.8	0.874
Birth weight (g) of the second twin	2402.5 ± 332.8	2389.4 ± 374.6	2419.3 ± 298.8	0.634
1 min Apgar of the first twin	9.4 ± 0.9	9.6 ± 0.7	9.1 ± 1.1	0.373
1 min Apgar of the second twin	8.9 ± 1.2	8.9 ± 1.3	8.9 ± 1.2	0.912
5 min Apgar of the first twin	9.7 ± 0.8	9.8 ± 0.4	9.6 ± 1.1	0.816
5 min Apgar of the second twin	9.8 ± 0.4	9.9 ± 0.3	9.7 ± 0.5	0.39
5 min Apgar < 7 of the first twin	0/16 (0.0 (0.0–20.6))	0/9 (0.0 (0.0–33.6))	0/7 (0.0 (0.0–41.0))	1.0
5 min Apgar < 7 of the second twin	0/16 (0.0 (0.0–20.6))	0/9 (0.0 (0.0–33.6))	0/7 (0.0 (0.0–41.0))	1.0
Umbilical artery pH of the first twin	7.3 ± 0.1	7.3 ± 0.0	7.2 ± 0.0	0.002
Umbilical artery pH of the second twin	7.3 ± 0.1	7.3 ± 0.0	7.2 ± 0.1	0.345
Umbilical artery pH < 7.1 of the first twin	0/15 (0.0 (0.0–21.8))	0/9 (0.0 (0.0–33.6))	0/6 (0.0 (0.0–45.9))	1.0
Umbilical artery pH < 7.1 of the second twin	0/15 (0.0 (0.0–21.8))	0/9 (0.0 (0.0–33.6))	0/6 (0.0 (0.0–45.9))	1.0
Umbilical venous pH of the first twin	7.3 ± 0.1	7.3 ± 0.0	7.3 ± 0.1	0.022
Umbilical venous pH of the second twin	7.3 ± 0.1	7.3 ± 0.0	7.3 ± 0.1	0.123
Neonatal morbidity ** of the first twin	2/16 (12.5 (1.6–38.3))	0/9 (0.0 (0.0–33.6))	2/7 (28.6 (3.7–71.0))	0.175
Neonatal morbidity ** of the second twin	2/16 (12.5 (1.6–38.3))	1/9 (11.1 (0.3–48.2))	1/7 (14.3 (0.4–57.9))	1.0
Neonatal death	0/16 (0.0 (0.0–20.6))	0/9 (0.0 (0.0–33.6))	0/7 (0.0 (0.0–41.0))	1.0
Positive RT-PCR SARS-CoV-2, first twin	1/6 (16.7 (0.4–64.1))	0/2 (0.0 (0.0–84.2))	1/4 (25.0 (0.6–80.6))	1.0
Positive RT-PCR SARS-CoV-2, second twin	1/3 (33.3 (0.8–90.6))	0/0 (0.0 (0.0–0.0))	1/3 (33.3 (0.8–90.6))	1.0
Positive cord-blood SARS-CoV-2 immunoglobulin G antibodies, first twin	1/5 (20.0 (0.5–71.6))	0/2 (0.0 (0.0–84.2))	1/3 (33.3 (0.8–90.6))	1.0
Positive cord-blood SARS-CoV-2 immunoglobulin G antibodies, second twin	2/3 (66.7 (9.4–99.2))	1/1 (100.0 (2.5–100.0))	1/2 (50.0 (1.3–98.7))	1.0

Data are given as n/N (% (95% CI)) or mean ± SD. * Defined as at least one of the following: admission to the intensive care unit, intubation, or extracorporeal membrane oxygenation. ** Including sepsis of unknown origin, neonatal jaundice, anemia, congenital anomalies, neonatal thrombocytopenia, and others.

**Table 3:** Odds ratios (ORs), adjusted odds ratios (aORs), and 95% confidence intervals (CIs) for perinatal outcomes among the symptomatic and asymptomatic pregnant patients with SARS-CoV-2 infection.

Variable	OR (95% CI)	aOR (95% CI)
Preterm birth	0.742 (0.382–1.443)	0.745 (0.313–1.776)
Fetal growth restriction	1.148 (0.410–3.212)	0.904 (0.245–3.340)
Preeclampsia	0.683 (0.095–4.892)	1.204 (0.103–14.074)
Stillbirth	0.696 (0.043–11.190)	0.925 (0.054–15.962)
Cesarean section	1.107 (0.726–1.689)	1.060 (0.630–1.782)
Umbilical artery pH < 7.1	1.519 (0.303–7.611)	1.217 (0.158–9.357)
Neonatal morbidity *	1.141 (0.606–2.148)	0.914 (0.447–1.868)

aOR adjusted for nulliparity, pre-existing chronic diseases, maternal age > 36 years old, obesity, and smoking habit. * Including sepsis of unknown origin, neonatal jaundice, anemia, congenital anomalies, neonatal thrombocytopenia, and others.

**Table 4:** Characteristics of 487 pregnant women with SARS-CoV-2 infection, overall and according to the vaccination status.

	Total Sample (n = 487)	Vaccinated (n = 201)	Not Vaccinated (n = 286)	*p*
Maternal age (years)	32.0 ± 5.5	33.0 ± 4.9	31.2 ± 5.8	<0.001
Pre-existing chronic disease *	93/486 (19.1 (15.7–22.9))	44/201 (21.9 (16.4–28.3))	49/285 (17.2 (13.0–22.1))	0.2
Smoking habit	32/485 (6.6 (4.6–9.2))	12/199 (6.0 (3.2–10.3))	20/286 (7.0 (4.3–10.6))	0.714
Weight (kg)	66.4 ± 14.1	65.3 ± 13.6	67.4 ± 14.5	0.118
Obesity **	47/338 (13.9 (10.4–18.1))	19/159 (11.9 (7.4–18.0))	28/179 (15.6 (10.7–21.8))	0.349
Nulliparity	240/483 (49.7 (45.1–54.2))	105/198 (53.0 (45.8–60.1))	135/285 (47.4 (41.5–53.3))	0.23
Twin pregnancy	16/487 (3.3 (1.9–5.3))	5/201 (2.5 (0.8–5.7))	11/286 (3.8 (1.9–6.8))	0.452
Assisted reproductive techniques	36/487 (7.4 (5.2–10.1))	24/201 (11.9 (7.8–17.2))	12/286 (4.2 (2.2–7.2))	0.002
Artificial insemination	2/487 (0.4 (0.0–1.5))	2/201 (1.0 (0.1–3.5))	0/286 (0.0 (0.0–1.3))	0.17
In vitro fertilization	34/487 (7.0 (4.9–9.6))	22/201 (10.9 (7.0–16.1))	12/286 (4.2 (2.2–7.2))	0.006
Egg donation	4/487 (0.8 (0.2–2.1))	3/201 (1.5 (0.3–4.3))	1/286 (0.3 (0.0–1.9))	0.311
SARS-CoV-2 infection during 1st trimester	59/487 (12.1 (9.4–15.3))	11/201 (5.5 (2.8–9.6))	48/286 (16.8 (12.6–21.6))	<0.001
SARS-CoV-2 infection during 2nd trimester	149/487 (30.6 (26.5–34.9))	74/201 (36.8 (30.1–43.9))	75/286 (26.2 (21.2–31.7))	0.016
SARS-CoV-2 infection during 3rd trimester	279/487 (57.3 (52.8–61.7))	116/201 (57.7 (50.6–64.6))	163/286 (57.0 (51.0–62.8))	0.926
Symptomatic SARS-CoV-2 infection	287/487 (58.9 (54.4–63.3))	125/201 (62.2 (55.1–68.9))	162/286 (56.6 (50.7–62.5))	0.226

Data are given as n/N (%) or mean ± SD. * Including diabetes, chronic hypertension, asthma, autoimmune disease, infection by human immunodeficiency virus, hepatitis B virus, or hepatitis C virus. ** Defined as body mass index ≥ 30 kg/m^2^.

**Table 5:** Maternal and perinatal outcomes of 487 pregnancies with SARS-CoV-2 infection, overall and according to the vaccination status.

	Total Sample (n = 487)	Vaccinated (n = 201)	Not Vaccinated (n = 286)	*p*
**Maternal outcome**				
Pneumonia	28/487 (5.7 (3.9–8.2))	4/201 (2.0 (0.5–5.0))	24/286 (8.4 (5.5–12.2))	0.003
Hospital admission due to SARS-CoV-2 infection	27/487 (5.5 (3.7–8.0))	4/201 (2.0 (0.5–5.0))	23/286 (8.0 (5.2–11.8))	0.004
Admission into the intensive care unit	11/27 (40.7 (22.4–61.2))	1/4 (25.0 (0.6–80.6))	10/23 (43.5 (23.2–65.5))	0.624
Composite adverse maternal outcome *	11/487 (2.3 (1.1–4.0))	1/201 (0.5 (0.0–2.7))	10/286 (3.5 (1.7–6.3))	0.031
Antibiotic treatment	13/487 (2.7 (1.4–4.5))	1/201 (0.5 (0.0–2.7))	12/286 (4.2 (2.2–7.2))	0.019
Corticosteroid drug	14/487 (2.9 (1.6–4.8))	1/201 (0.5 (0.0–2.7))	13/286 (4.5 (2.4–7.6))	0.01
Antiviral drug	10/487 (2.1 (1.0–3.7))	1/201 (0.5 (0.0–2.7))	9/286 (3.1 (1.4–5.9))	0.052
Oxygen therapy	16/487 (3.3 (1.9–5.3))	1/201 (0.5 (0.0–2.7))	15/286 (5.2 (3.0–8.5))	0.003
Intubation	7/487 (1.4 (0.6–2.9))	1/201 (0.5 (0.0–2.7))	6/286 (2.1 (0.8–4.5))	0.248
Extracorporeal membrane oxygenation	1/487 (0.2 (0.0–1.1))	0/201 (0.0 (0.0–1.8))	1/286 (0.3 (0.0–1.9))	1.0
**Perinatal outcome**				
Ectopic pregnancy	1/487 (0.2 (0.0–1.1))	0/201 (0.0 (0.0–1.8))	1/286 (0.3 (0.0–1.9))	1.0
Miscarriage	9/487 (1.8 (0.8–3.5))	3/201 (1.5 (0.3–4.3))	6/286 (2.1 (0.8–4.5))	0.742
Stillbirth	2/487 (0.4 (0.0–1.5))	2/201 (1.0 (0.1–3.5))	0/286 (0.0 (0.0–1.3))	0.17
***Completed pregnancies (excluding ectopic pregnancy and miscarriage)***				
Threatened preterm labor	5/477 (1.0 (0.3–2.4))	2/198 (1.0 (0.1–3.6))	3/279 (1.1 (0.2–3.1))	1.0
Small for gestational age	4/477 (0.8 (0.2–2.1))	4/198 (2.0 (0.6–5.1))	0/279 (0.0 (0.0–1.3))	0.029
Fetal growth restriction	16/477 (3.4 (1.9–5.4))	6/198 (3.0 (1.1–6.5))	10/279 (3.6 (1.7–6.5))	0.802
Gestational diabetes mellitus	30/477 (6.3 (4.3–8.9))	11/198 (5.6 (2.8–9.7))	19/279 (6.8 (4.1–10.4))	0.703
Gestational hypertension	6/477 (1.3 (0.5–2.7))	1/198 (0.5 (0.0–2.8))	5/279 (1.8 (0.6–4.1))	0.408
Preeclampsia	4/477 (0.8 (0.2–2.1))	1/198 (0.5 (0.0–2.8))	3/279 (1.1 (0.2–3.1))	0.645
Preterm premature rupture of membranes	3/477 (0.6 (0.1–1.8))	2/198 (1.0 (0.1–3.6))	1/279 (0.4 (0.0–2.0))	0.573
Gestational age at delivery (weeks)	39.2 ± 1.7	39.2 ± 1.6	39.2 ± 1.8	0.507
Cesarean section	121/477 (25.4 (21.5–29.5))	59/198 (29.8 (23.5–36.7))	62/279 (22.2 (17.5–27.6))	0.07
Preterm birth	38/475 (8.0 (5.7–10.8))	13/196 (6.6 (3.6–11.1))	25/279 (9.0 (5.9–12.9))	0.395
*Singleton pregnancy*				
Gestational age at delivery (weeks)	39.3 ± 1.7	39.3 ± 1.6	39.3 ± 1.8	0.343
Preterm birth	29/459 (6.3 (4.3–8.9))	11/191 (5.8 (2.9–10.1))	18/268 (6.7 (4.0–10.4))	0.846
Birth weight (g)	3254.2 ± 480.6	3236.3 ± 424.2	3267.0 ± 517.5	0.308
1 min Apgar	9.2 ± 1.1	9.2 ± 0.9	9.2 ± 1.2	0.349
5 min Apgar	9.9 ± 0.5	9.9 ± 0.5	9.9 ± 0.5	0.04
5 min Apgar < 7	2/458 (0.4 (0.1–1.6))	1/190 (0.5 (0.0–2.9))	1/268 (0.4 (0.0–2.1))	1.0
Umbilical artery pH	7.2 ± 0.1	7.2 ± 0.1	7.2 ± 0.1	0.976
Umbilical artery pH < 7.1	6/447 (1.3 (0.5–2.9))	3/186 (1.6 (0.3–4.6))	3/261 (1.1 (0.2–3.3))	0.697
Umbilical venous pH	7.3 ± 0.1	7.3 ± 0.1	7.3 ± 0.1	0.23
Neonatal morbidity **	43/459 (9.4 (6.9–12.4))	25/191 (13.1 (8.7–18.7))	18/268 (6.7 (4.0–10.4))	0.023
Neonatal death	1/459 (0.2 (0.0–1.2))	0/191 (0.0 (0.0–1.9))	1/268 (0.4 (0.0–2.1))	1.0
Positive RT-PCR SARS-CoV-2	2/122 (1.6 (0.2–5.8))	0/28 (0.0 (0.0–12.3))	2/94 (2.1 (0.3–7.5))	1.0
Positive cord-blood SARS-CoV-2 immunoglobulin G antibodies	52/84 (61.9 (50.7–72.3))	1/1 (100.0 (2.5–100.0))	51/83 (61.4 (50.1–71.9))	1.0
*Twin pregnancy*				
Gestational age at delivery (weeks)	36.4 ± 0.9	36.6 ± 0.6	36.3 ± 1.0	0.278
Preterm birth	9/16 (56.3 (29.9–80.2))	2/5 (40.0 (5.3–85.3))	7/11 (63.6 (30.8–89.1))	0.596
Birth weight (g) of the first twin	2408.6 ± 363.0	2398.0 ± 352.8	2413.4 ± 384.4	0.777
Birth weight (g) of the second twin	2402.5 ± 332.8	2339.0 ± 309.3	2431.4 ± 353.5	0.61
1 min Apgar of the first twin	9.4 ± 0.9	9.4 ± 0.9	9.4 ± 0.9	0.949
1 min Apgar of the second twin	8.9 ± 1.2	7.8 ± 1.3	9.4 ± 0.8	0.021
5 min Apgar of the first twin	9.7 ± 0.8	9.6 ± 0.5	9.7 ± 0.9	0.212
5 min Apgar of the second twin	9.8 ± 0.4	9.6 ± 0.5	9.9 ± 0.3	0.155
5 min Apgar < 7 of the first twin	0/16 (0.0 (0.0–20.6))	0/5 (0.0 (0.0–52.2))	0/11 (0.0 (0.0–28.5))	1.0
5 min Apgar < 7 of the second twin	0/16 (0.0 (0.0–20.6))	0/5 (0.0 (0.0–52.2))	0/11 (0.0 (0.0–28.5))	1.0
Umbilical artery pH of the first twin	7.3 ± 0.1	7.3 ± 0.0	7.3 ± 0.1	0.387
Umbilical artery pH of the second twin	7.3 ± 0.1	7.3 ± 0.1	7.3 ± 0.0	0.713
Umbilical artery pH < 7.1 of the first twin	0/15 (0.0 (0.0–21.8))	0/5 (0.0 (0.0–52.2))	0/10 (0.0 (0.0–30.8))	1.0
Umbilical artery pH < 7.1 of the second twin	0/15 (0.0 (0.0–21.8))	0/5 (0.0 (0.0–52.2))	0/10 (0.0 (0.0–30.8))	1.0
Umbilical venous pH of the first twin	7.3 ± 0.1	7.3 ± 0.0	7.3 ± 0.1	0.776
Umbilical venous pH of the second twin	7.3 ± 0.1	7.3 ± 0.1	7.3 ± 0.1	0.776
Neonatal morbidity ** of the first twin	2/16 (12.5 (1.6–38.3))	1/5 (20.0 (0.5–71.6))	1/11 (9.1 (0.2–41.3))	1.0
Neonatal morbidity ** of the second twin	2/16 (12.5 (1.6–38.3))	0/5 (0.0 (0.0–52.2))	2/11 (18.2 (2.3–51.8))	1.0
Neonatal death	0/16 (0.0 (0.0–20.6))	0/5 (0.0 (0.0–52.2))	0/11 (0.0 (0.0–28.5))	1.0
Positive RT-PCR SARS-CoV-2, first twin	1/6 (16.7 (0.4–64.1))	0/0 (0.0 (0.0–0.0))	1/6 (16.7 (0.4–64.1))	1.0
Positive RT-PCR SARS-CoV-2, second twin	1/3 (33.3 (0.8–90.6))	0/0 (0.0 (0.0–0.0))	1/3 (33.3 (0.8–90.6))	1.0
Positive cord-blood SARS-CoV-2 immunoglobulin G antibodies, first twin	1/5 (20.0 (0.5–71.6))	0/0 (0.0 (0.0–0.0))	1/5 (20.0 (0.5–71.6))	1.0
Positive cord-blood SARS-CoV-2 immunoglobulin G antibodies, second twin	2/3 (66.7 (9.4–99.2))	0/0 (0.0 (0.0–0.0))	2/3 (66.7 (9.4–99.2))	1.0

Data are given as n/N (% (95% CI)) or mean ± SD. * Defined as at least one of the following: admission to the intensive care unit, intubation, or extracorporeal membrane oxygenation. ** Including sepsis of unknown origin, neonatal jaundice, anemia, congenital anomalies, neonatal thrombocytopenia, and others.

**Table 6:** Odds ratios (ORs), adjusted odds ratios (aORs), and 95% confidence intervals (CIs) for perinatal outcomes among the vaccinated and unvaccinated pregnant women with SARS-CoV-2 infection.

Variable	OR (95% CI)	aOR (95% CI)
Pneumonia	0.222 (0.076–0.649)	0.209 (0.044–0.985)
Hospital admission due to COVID-19	0.232 (0.079–0.682)	0.209 (0.044–0.985)
Preterm birth	0.722 (0.360–1.449)	1.024 (0.433–2.424)
Fetal growth restriction	0.841 (0.300–2.352)	1.047 (0.289–3.787)
Preeclampsia	0.467 (0.048–4.523)	0.517 (0.045–5.940)
Cesarean section	1.486 (0.981–2.250)	1.200 (0.723–1.993)
Umbilical artery pH < 7.1	0.701 (0.140–3.513)	0.893 (0.120–6.626)
Neonatal morbidity *	2.093 (1.123–3.900)	1.982 (0.957–4.103)

aOR adjusted for nulliparity, pre-existing chronic diseases, maternal age > 36 years old, obesity, and smoking habit. * Including sepsis of unknown origin, neonatal jaundice, anemia, congenital anomalies, neonatal thrombocytopenia, and others.

## Data Availability

The data in this study were obtained from the clinical program of the University and Polytechnic Hospital La Fe. Such a dataset may be completely available on request to the corresponding author. The data are not publicly available due to privacy.
